# Fr-TM-align: a new protein structural alignment method based on fragment alignments and the TM-score

**DOI:** 10.1186/1471-2105-9-531

**Published:** 2008-12-12

**Authors:** Shashi Bhushan Pandit, Jeffrey Skolnick

**Affiliations:** 1 Center for the Study of Systems Biology, School of Biology, Georgia Institute of Technology, Atlanta, USA

## Abstract

**Background:**

Protein tertiary structure comparisons are employed in various fields of contemporary structural biology. Most structure comparison methods involve generation of an initial seed alignment, which is extended and/or refined to provide the best structural superposition between a pair of protein structures as assessed by a structure comparison metric. One such metric, the TM-score, was recently introduced to provide a combined structure quality measure of the coordinate root mean square deviation between a pair of structures and coverage. Using the TM-score, the TM-align structure alignment algorithm was developed that was often found to have better accuracy and coverage than the most commonly used structural alignment programs; however, there were a number of situations when this was not true.

**Results:**

To further improve structure alignment quality, the Fr-TM-align algorithm has been developed where aligned fragment pairs are used to generate the initial seed alignments that are then refined using dynamic programming to maximize the TM-score. For the assessment of the structural alignment quality from Fr-TM-align in comparison to other programs such as CE and TM-align, we examined various alignment quality assessment scores such as PSI and TM-score. The assessment showed that the structural alignment quality from Fr-TM-align is better in comparison to both CE and TM-align. On average, the structural alignments generated using Fr-TM-align have a higher TM-score (~9%) and coverage (~7%) in comparison to those generated by TM-align. Fr-TM-align uses an exhaustive procedure to generate initial seed alignments. Hence, the algorithm is computationally more expensive than TM-align.

**Conclusion:**

Fr-TM-align, a new algorithm that employs fragment alignment and assembly provides better structural alignments in comparison to TM-align. The source code and executables of Fr-TM-align are freely downloadable at: .

## Background

Protein tertiary structure comparison is widely employed in the field of structural biology, with applications ranging from protein fold classification [1-3], protein structure prediction and modeling [4-8] to structure-based protein function annotation [9-11]. With the rapid increase in the number of protein structures deposited in the Protein Data Bank (PDB) [12], it is important to develop faster and better algorithms to compare protein structures. In general, there are two types of protein tertiary structure comparisons approaches. The easiest involves the comparison of a pair of protein structures with an a priori specified equivalence between pairs of residues (as provided by sequence or threading alignments [13]). The second type involves the comparison when the set of equivalent residues is not a priori given. Therefore, an optimal structural alignment needs to be identified; this problem is NP-hard with no exact solution [14]. Nevertheless, a number of methods have been developed that employ heuristics to search for the best structural alignment. These methods use different representations of protein structure, definitions of similarity measures and optimization algorithms [15,16]. Some approaches compare the respective distance matrices of each protein structure, trying to minimize the intra-atomic distances for the set of aligned substructures [17-21]; this is the approach employed in the widely used DALI algorithm [18]. Another approach, tries to minimize the inter-atomic distances between two structures [22-28]; representatives of this type of algorithm include CE [26], MAMMOTH [28], and TM-align [22].

In practice, most structural alignment procedures start with the generation of an initial set of equivalent residues. Then, using a structural similarity score, the initial seed alignment is extended and/or refined using methods such as dynamic programming or Monte Carlo procedures. Finally, the structural similarity score is assessed; this is generally done on the basis of the statistical significance of the structural similarity. In practice, many structural alignment algorithms use fragment assembly to build the initial set of equivalences [18-20,26,28-30]. This involves the comparison of many, if not all, small fragments in the two structures. Then, similar fragments are assembled into a larger, consistent set. Fragments can be secondary structure elements [20,30] or arbitrary substructures of a given length as in CE [26] or MAMMOTH [28].

In most structural alignment methods, structural similarity is assessed by a structure comparison score. One commonly used measure is the root-mean-square deviation, RMSD, between a pair of structures with a specified set of equivalent residues. As pointed out by Zhang and Skolnick [31] among others [32], statistically significant values of the RMSD are length dependent. Another problem is that the RMSD can be reduced by decreasing the coverage (the number of aligned residues). These issues were addressed by introduction of the TM-score [31], which is a modification of the Levitt-Gerstein (LG) weight factor [33] that weights residue pairs at smaller distances greater than those at larger distances. The TM-score is length independent, with a value of 0.30 (0.01) for the average (standard deviation of the) TM-score of the best structural alignment for randomly selected pairs of proteins [34].

Based on the TM-score, a new structural alignment algorithm TM-align [22] was developed. TM-align employs a very simple approach that uses both gapless threading and secondary structure similarity to generate the initial set of equivalent residues. This set of aligned residues is refined using dynamic programming to maximize the TM-score. The scoring matrix used for dynamic programming is derived from the TM-score rotation matrix, which results in faster convergence and a better structural alignment. On average, TM-align provides structural alignments with higher accuracy and coverage than the most often-used methods such as DALI and CE. In a separate study [35], TM-align was compared with other competitive structural alignment programs using different measures of structural similarity, with the result that TM-align is one of the best structural alignment programs [22,35]. However, TM-align sometimes is unable to identify a good structural similarity. To address this issue, in the present work, we improve the TM-align program by generating a better initial set of equivalences using a fragment assembly approach. This is followed by heuristic iterations involving the TM-score rotation matrix and dynamic programming to obtain the structural alignment with the largest TM-score.

## Methods

### Datasets

We have used two datasets for the evaluation of different structural alignment programs. The first dataset is the same as that used in the original TM-align paper [22] and consists of 200 proteins that range in size from 46 to 1058 residues. The first dataset is also used in deriving various parameters for Fr-TM-align algorithm. The second dataset has 200 proteins, and is a randomly selected, representative subset of the PDB template library of non-homologous proteins with pairwise sequence identity of ≤ 35% [13]. It is comprised of proteins whose length ranges from 40 to 910 residues and includes representatives of all secondary structure classes, *viz*. all α, all β or mixed α/β proteins.

### Algorithm and implementation

Fr-TM-align employs the backbone C_α _coordinates of the protein structures for the structural alignment. The Fr-TM-align algorithm consists of the following steps:

#### 1. Generation and scoring of aligned fragment pairs

In the first step, an optimal superposition between the fragments from two protein structures is obtained that maximizes the structural similarity score as given by the GL-score, defined as:

(1) $$GL-score={\sum_{i=1}^{L_{F}}\left(1+\frac{d_{i}^{2}}{d_{a}^{2}}\right)}^{-1}$$

where *d*_*a *_= 0.5 and L_F _is the length of the fragment. Here, the value of *d*_*a *_is derived empirically. In order to derive the empirical value of *d*_*a*_, we have used four reasonable values for *d*_*a *_= 0.25, 0.5, 1.0 or 1.5, and the value which resulted in the maximum average TM-score for all pairs of alignments in the training dataset using Fr-TM-align was selected. In addition, the secondary structure similarity between the two fragments is given by the SS-score, which is simply the number of residues with identical secondary structure normalized by the length of the fragment. The secondary structure assignment procedure is same as in TM-align [22].

Let *L*_*A *_and *L*_*B *_be the length of the first and second chains respectively, and let *L*_*F *_be the length of the fragment. Then, the numbers of fragments are *I *= *L*_*A*_/*L*_*F *_and *J *= *L*_*B*_/*L*_*F*_. The length of the fragment is selected empirically. *L*_*F*_, is 8 residues, if the length of the smaller protein is less than 100 residues; otherwise, *L*_*F *_= 12 residues. We perform an all-against-all (*I ***X ***J*) structural superposition of the non-overlapping fragments from the two protein structures. The structural similarity and secondary structure similarity of the fragments from the two proteins as given by their GL-score and SS-score are stored in the G(I, J) and S(I, J) scoring matrices respectively.

#### 2. Assembly of fragments and generation of seed alignments

In order to generate different seed alignments, the fragments are assembled into a large consistent set. For this, we employ dynamic programming (DP) with no end gap penalty to align fragments using the scoring matrices derived from the fragment comparison in the first step. We observed that the optimal fragment alignment does not usually result in the structural alignment with highest TM-score. Hence, in addition to the optimal alignment obtained using DP, we generate three suboptimal alignments using a variation of the Waterman and Eggert algorithm [36]. The generation of suboptimal alignments involves re-computing of the forward trace matrix for finding the optimal path with the modification that the similarity score of the previously aligned positions is not used in the calculation of the forward trace matrix. The optimal path alignment is obtained by back tracing from the last column/row using this forward trace matrix using no end gap penalty. Other variations of the DP algorithm to generate the suboptimal alignment [37] would have been equally applicable.

We have used two scoring matrices for DP, G(I, J) and (G(I, J)+S(I, J))/2, along with three different gap opening penalties of -0.6, -0.1 and 0.0, which are chosen empirically to generate various fragment alignments. The fragment alignment, in turn, provides the initial set of equivalent residues between the two protein structures. We could use these initial set of alignments as seed alignments and use heuristic iteration to get best structural alignment. However, we observed that refining the alignment with another round of DP before heuristic iteration results in alignments with improved TM-score.

The fragment alignment is used to generate initial equivalent residues, which are modified by DP using three different scoring matrices to form the final set of seed alignments. The three scoring matrices are: (1) a distance score matrix generated by rotating one of the structures by the RMSD rotation matrix based on the aligned residues. The RMSD rotation matrix is the matrix which minimizes the distance between pairs of equivalent residues for given two structures. This provides the best structural superposition (lowest RMSD) of the predefined equivalent residues between two structures and is used to calculate the distance matrix (as defined in equation 2(a)), which is used in the DP step. (2) Modification of the first scoring matrix based on the identity of the secondary structure assignment of the residues. When the secondary structure assignment of the pair of aligned residues is the same, 0.5 is added to the respective score. (3) A distance score matrix generated using the TM-score rotation matrix based on the aligned residues. Along with these scoring matrices, we have used gap opening penalties of -1.0 and 0.0. This step is repeated for each fragment alignment. Finally, this gives a set of seed alignments, which are refined using the heuristic iteration procedure discussed below. The generation of initial seed alignments is an exhaustive process. However, we can generate fewer seed alignments by using only one gap opening penalty rather then using two. This decreases the computational time of the algorithm. We have implemented this in the faster version of the program (see Results section).

#### 3. Heuristic Iteration

The above mentioned initial alignments are submitted to heuristic iteration as described in TM-align [22]. In this procedure, we first rotate the structures by the TM-score rotation matrix (the rotation matrix, which provides the best TM-score after superposition for a set of equivalent residues) based on the aligned residues in the initial alignment. This rotation matrix is identified in the process of finding the set of equivalent residues that gives maximum TM-score. The score similarity matrix is defined as:

(2a) $$\text{S(k,l) }=\frac{1}{1+d_{kl}^{2}/d_{0}{\left(L_{\min}\right)}^{2}}\;$$

where

(2b) $$d_{0}(L_{\min})=1.24\sqrt[{3}]{L_{\min}-15}-1.8$$

Here, *d*_*kl *_is the distance of the *k*^th ^residue in structure 1 from the *l*^th ^residue in structure 2 under the TM-score superposition, with *L*_*min *_the length of the smaller protein. A new alignment can be obtained by implementing DP on the matrix S(k, l), with optimal gap opening penalties of -0.6 and 0.0; these parameters were chosen empirically (see following paragraph). We then superimpose the structures by the TM-score rotation matrix according to the new alignment and obtain a newer alignment by implementing DP with the new score matrix. The procedure is repeated until the alignment becomes stable and the alignment with the highest TM-score is returned.

In all the above mentioned DP procedures, we obtained gap opening penalties with the objective function being the maximization of the average TM-score of the final alignment for all pairs of proteins in the training dataset. For this, we spanned gap penalties from -1.6 to 0.0 with a bin width of 0.1. The gap opening penalty value selected was the one that resulted in the maximum average TM-score of the final alignment for all pairs of proteins in the training dataset.

### Alignment quality assessment

There are various metrics used in the literature to objectively assess structural alignment quality [38]. However, most involve normalization of the RMSD by the number of aligned residues and protein length. Hence, the metric is not completely independent of the lengths of proteins being aligned. Here, we have used a geometric match metric, the TM-score, for the objective assessment of the structural alignment quality from various structural alignment programs. This metric provides a balance between coverage and alignment accuracy (low RMSD). The TM-score is defined as:

(3) TM−score=Max[1Ltarget∑i=1Lali(1+(did0(Ltarget))2)−1]

Here, *L*_target _is the length of the target protein that other PDB structures are aligned to; *L*_*ali *_is the number of aligned residues; *d*_*i *_is the distance between the *i*th pair of aligned residues and $d_{0}(L_{t\arg et})=1.24\sqrt[{3}]{L_{\text{target}}-15}-1.8$, which is the average distance between a pair of residues for the best structural alignment in a randomly selected pair of structures of length *L*_target_.

In addition, we have used other scores for the objective structural alignment quality assessment and for the comparison of structural alignment algorithms. The percentage of structural similarity (PSI) is defined as the number of aligned amino acid pairs with C_α _atoms that are closer in space then 4 Å after optimal superposition normalized by the length of the shorter chain in the alignment. The relevant PSI (rPSI) value does not include fragments shorter than four aligned amino acid from the calculated PSI value. The coordinate RMSD (cRMSD) is computed for all aligned pairs after optimal superposition. The cRMSD (core) is computed for those aligned pairs that contribute to the PSI value [35]. Here, PSI/rPSI provides a more detailed view of the alignment. However, these values are length dependent.

## Results and discussion

In the literature, various structural alignment methods have been evaluated in different ways [38]. Most evaluations use SCOP [1] or CATH [2] as the gold standard and assess the structural alignment based on the fold classifications found in these databases [35,39-41]. Because the SCOP and CATH classifications are discrete, a drawback in this kind of evaluation is that the detailed alignment quality is not taken into account. Moreover, evolutionary information is also used in the SCOP or CATH classification apart from the structure of the protein under consideration. Recent studies [42,43] have shown that significant structural similarity exists between proteins belonging to different fold families in the CATH and SCOP classifications. Here, we have compared different structural alignments pairs of structures purely by their geometric match.

### Comparison of alignment quality

In the present analysis, we have evaluated the performance of the structural alignments algorithms CE, TM-align and Fr-TM-align on two different datasets (as described in the Methods). We have compared the alignment quality of Fr-TM-align with other structural alignment algorithms using cRMSD, TM-score, PSI and rPSI. These measures of alignment accuracy capture various features of the alignment. For example, PSI counts spatially close residues, whereas rPSI represents core continuous fragments that are spatially close. The TM-score represents a quality measure that combines both alignment coverage and accuracy.

Table 1 shows the summary of structural alignments of 200 × 199 non-homologous protein pairs from the two datasets that are obtained from CE, TM-align and Fr-TM-align. Since the TM-score depends on the length of the target, we count all the comparisons with respect to both partners in Table 1. Dataset 1 was used for the optimization of Fr-TM-align step of fragment selection and assembly. Dataset 2 is used as a testing set.

**Table 1:** Structural alignment by different algorithms for two datasets

**Average over all pairs (39,800)**^a^							
	**⟨cRMSD⟩**	**⟨cR(core)⟩**	**⟨TM⟩**	**⟨PSI %⟩**	**⟨rPSI %⟩**	**⟨L⟩**	**⟨cov %⟩**
*Dataset 1*							
CE	5.7 (1.1)	2.8 (0.2)	0.185 (0.070)	18.9 (10.3)	8.4 (8.3)	69.9	37.2
TM-align	5.1 (1.0)	2.5 (0.3)	0.255 (0.087)	29.5 (11.1)	17.7 (11.4)	87.8	42.3
Fr-TM-align	5.0 (1.0)	2.5 (0.3)	0.279 (0.091)	33.1 (12.0)	20.0 (12.3)	93.8	45.3
							
*Dataset 2*							
CE	5.7 (1.1)	2.8 (0.2)	0.190 (0.073)	21.5 (11.7)	10.5 (10.1)	65.8	39.7
TM-align	4.8 (1.1)	2.4 (0.4)	0.256 (0.093)	32.3 (11.5)	21.0 (12.3)	77.1	42.1
Fr-TM-align	4.7 (1.0)	2.4 (0.4)	0.280 (0.097)	35.9 (12.2)	23.1 (13.1)	82.6	45.2

^a ^Results are averaged over all structure pairs. cRMSD, cR(core), L, cov, TM, PSI and rPSI denotes coordinate RMSD (in Å), cRMSD (in Å) for all aligned pairs that contribute to PSI, number of aligned residues, coverage of aligned residues, TM-score, percentage structural similarity and relevant percentage structural similarity (see method section). The number in parenthesis is the standard deviation.

In Table 1, columns 2–6 show the alignment accuracy as measured by various scores and columns 7–8 show the number of aligned residues and coverage averaged over 39,800 protein pairs. Based on cRMSD, the structural alignments from Fr-TM-align have on average better accuracy in comparison to both CE and TM-align for both datasets. Similarly, cRMSD (core) is also better on average for both TM-align and Fr-TM-align in comparison to CE. However, cRMSD (core) shows no difference between Fr-TM-align and TM-align. Based on another alignment accuracy score PSI or rPSI, Fr-TM-align performs better in comparison to both CE and TM-align for both the datasets. All three programs show a significant decreased value in the value of rPSI in comparison to PSI. This shows that all three programs achieve high PSI value by finding aligned fragments of length less than four residues in length.

Based on another structural alignment accuracy measure (TM-score), Fr-TM-align resulted in structural alignments with higher TM-score in comparison to both TM-align and CE. The higher TM-score could result from better identifying structurally similar regions with/without including more residues in the aligned region or by decreasing the number of aligned residues. As shown in Table 1, the average coverage and average number of aligned residues is higher for Fr-TM-align in comparison to TM-align and CE for both datasets. Hence, the improvement observed in TM-score by Fr-TM-align mainly results from the better identification of structurally similar regions in the pair of structures. This is also evident by the observed higher PSI/rPSI value for the alignments from Fr-TM-align. This suggests that Fr-TM-align better identifies structurally similar regions with increased accuracy and higher coverage. Next, we tested the statistical significance of the observed increase in the mean TM-score, PSI and rPSI for Fr-TM-align in comparison to TM-align and CE, using the unpaired t-test for the independent samples. The improvement in the mean TM-score, PSI and rPSI for Fr-TM-align is found to be statistically significant (p-value << 0.001).

While the data in Table 1 are averaged over all structure pairs, where the majority have different folds and low TM-score, a more realistic assessment is to evaluate the performance of different methods for the most significant match to a given target protein. Various methods employ different metrics of structural similarity: CE uses the CE z-score, whereas TM-align and Fr-TM-align use the TM-score. In the present analysis, we used both measures of structural similarity, the CE z-score and TM-score, to extract the most significant structurally similar protein pairs for the evaluation of the three methods.

In Table 2, for datasets 1 and 2, we analyzed protein pairs identified as being the most structurally similar as assessed by their CE z-score. For each protein in datasets 1 and 2, the protein pair with the highest CE z-score was selected from the CE alignments, and the structural alignment was generated by CE, TM-align and Fr-TM-align. It is evident from Table 2 that structural alignments from Fr-TM-align have better alignment accuracy than those from both CE and TM-align, when structural similarity is assessed by cRMSD. Moreover, on average, the Fr-TM-align generated alignments have higher coverage. Thus, using the TM-score as the metric for structural similarity, Fr-TM-align is able to better identify structurally similar regions in comparison to both CE and TM-align. This conclusion also holds when other measures of alignment quality (PSI/rPSI) are used.

**Table 2:** Comparison of best structural alignment (alignment with highest CE z-score) from CE

**Average of pairs with best z-score from CE (200 proteins for each dataset)^a^**							
	**⟨cRMSD_Z_⟩**	**⟨cR(core)_Z_⟩**	**⟨TM_Z_⟩**	**⟨PSI_Z _%⟩**	**⟨rPSI_Z _%⟩**	**⟨L_Z_⟩**	**⟨cov_Z _%⟩**
*Dataset 1*							
CE	4.0 (1.4)	2.4 (0.4)	0.403 (0.203)	47.5 (23.5)	36.9 (24.5)	118.5	57.8
TM-align	4.0 (1.2)	2.2 (0.4)	0.446 (0.194)	52.8 (21.2)	43.9 (23.9)	129.7	60.9
Fr-TM-align	3.9 (1.1)	2.2 (0.4)	0.459 (0.188)	54.7 (19.9)	45.7 (22.9)	132.6	62.3
							
*Dataset 2*							
CE	4.1 (1.4)	2.5 (0.4)	0.366 (0.170)	44.5 (22.4)	34.6 (23.1)	101.7	55.9
TM-align	4.1 (1.3)	2.3 (0.4)	0.408 (0.164)	48.5 (21.0)	39.5 (22.7)	112.4	58.8
Fr-TM-align	4.0 (1.3)	2.2 (0.4)	0.426 (0.161)	50.9 (20.4)	42.0 (22.5)	117.1	60.6

^a ^For each protein, we selected the protein pair with the highest z-score using the CE program. We used the same set of protein pairs selected from CE for comparison with the other programs. cRMSD_Z_, cR(core)_Z_, L_Z_, cov_Z_, TM_Z_, PSI_Z _and rPSI_Z _denotes coordinate RMSD (in Å), cRMSD (in Å) for all aligned pairs that contribute to PSI, number of aligned residues, coverage of aligned residues, TM-score, percentage structural similarity and relevant percentage structural similarity (see Methods). The number in parenthesis is the standard deviation.

Next, we used the TM-score to select the most structurally similar protein pairs. That is, for each protein in both datasets, we selected the template protein with the highest TM-score obtained by Fr-TM-align. Then, structural alignments between the selected pair of proteins are also generated by CE and TM-align, and the averages reported in Table 3. As is evident, structural alignments from Fr-TM-align have comparable or better accuracy in comparison to CE and TM-align when assessed by their cRMSD. Indeed, Fr-TM-align gives comparable or higher structural alignment accuracy as assessed by cRMSD, cRMSD (core), PSI and rPSI in comparison to both CE and TM-align.

**Table 3:** Comparison of best structural alignment (alignment with maximum TM-score) from Fr-TM-align

**Average of pairs with best TM-score from Fr-TM-align (200 proteins for each dataset)^a^**							
	**⟨cRMSD_M_⟩**	**⟨cR(core)_M_⟩**	**⟨TM_M_⟩**	**⟨PSI_M _%⟩**	**⟨rPSI_M _%⟩**	**⟨L_M_⟩**	**⟨cov_M _%⟩**
*Dataset 1*							
CE	4.5 (1.5)	2.5 (0.4)	0.392 (0.207)	39.1 (26.6)	29.3 (26.6)	120.9	59.2
TM-align	4.6 (1.7)	2.3 (0.4)	0.488 (0.163)	46.5 (24.5)	37.1 (27.2)	162.5	70.4
Fr-TM-align	4.5 (1.6)	2.3 (0.4)	0.522 (0.144)	50.0 (23.4)	40.4 (26.5)	170.6	74.5
							
*Dataset 2*							
CE	4.8 (1.5)	2.6 (0.4)	0.354 (0.175)	35.8 (24.2)	25.7 (24.1)	108.5	58.0
TM-align	4.7 (1.5)	2.3 (0.4)	0.451 (0.142)	43.9 (21.6)	34.1 (23.3)	139.3	67.5
Fr-TM-align	4.5 (1.5)	2.3 (0.4)	0.497 (0.127)	49.2 (22.0)	39.1 (24.4)	149.1	73.2

^a ^For each protein, we selected the protein pair with the highest TM-score using the Fr-TM-align program. We used the same set of protein pairs selected from Fr-TM-align for comparison with the other programs. cRMSD_M_, cR(core)_M_, L_M_, cov_M_, TM_M_, PSI_M _and rPSI_M _denotes coordinate RMSD (in Å), cRMSD (in Å) for all aligned pairs that contribute to PSI, number of aligned residues, coverage of aligned residues, TM-score, percentage structural similarity and relevant percentage structural similarity (See Methods). The number in parenthesis is the standard deviation.

For a more detailed analysis, we have mostly used the TM-score to compare the alignments from TM-align and Fr-TM-align. As discussed before, the TM-score provides an appropriate combined quality measure of coverage and accuracy (low RMSD). In addition, we have previously shown that the TM-score also has the strongest correlation with foldability using MODELLER [44], in comparison with other structural similarity scores [31].

### Comparison of TM-align and Fr-TM-align

**Figure 1:**
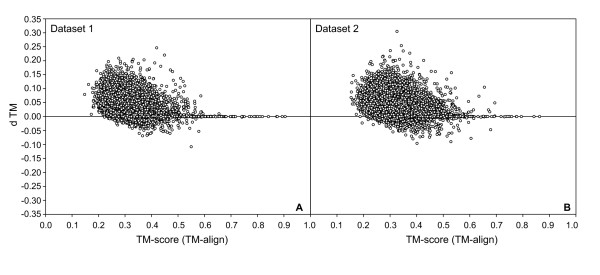
**A) Difference in TM-score from Fr-TM-align and TM-align plotted against the TM-score from TM-align for dataset 1.** B) Similar data as in A, but plotted for dataset 2. (dTM is defined as (TM-score (Fr-TM-align) – TM-score (TM-align)).

As shown in Table 1, on average, for both the training and testing datasets, Fr-TM-align resulted in alignments with higher TM-score (by ~9%) and higher average number of aligned residues (by ~7%) in comparison to TM-align. Here, we explore in detail the distribution of differences in TM-score between Fr-TM-align and TM-align. For a pair of protein structures, if Fr-TM-align results in a higher TM-score, when the second protein is used as target length, usually, it usually also results in a higher TM-score when the first protein is used for target length. Hence, in order to avoid double counting, we have considered only 19,900 pairs of structures with TM-score calculated with respect to the length of the smaller of the two proteins. Figures 1A and 1B shows the difference in TM-score between Fr-TM-align and TM-align plotted against the TM-score from TM-align for dataset 1 and dataset 2, respectively. As is evident from Figure 1, Fr-TM-align results in a better TM-score for most (~86%) pairs of structures. However, in a few cases (~7%), Fr-TM-align could not recover the structural alignment as given by TM-align.

**Figure 2:**
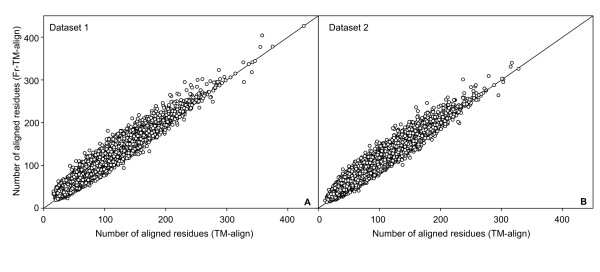
**A) Scatter plot showing the number of aligned residues from Fr-TM-align versus the number of aligned residues from TM-align for dataset 1.** B) Similar data as in A, but plotted for dataset 2.

**Figure 3:**
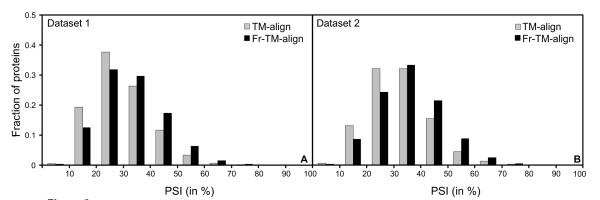
**A) Histogram showing the fraction of protein pairs in various PSI (in %) bins for the dataset 1.** B) Similar data as in A, but plotted for dataset 2.

Next, we compare the number of aligned residues. As shown in Figure 2, for most (~66%) protein pairs, Fr-TM-align results in a higher number of aligned residues. Hence, Fr-TM-align results in a higher TM-score with increased coverage. In addition to TM-score, we have also used PSI to compare alignment quality between TM-align and Fr-TM-align. Figures 3A and 3B show the frequency distribution of PSI (%) for the training and testing datasets respectively. Fr-TM-align identifies alignments with higher PSI in comparison to TM-align. In fact, Fr-TM-align assigns a PSI > 40% in ~27% of alignments, whereas TM-align is only able to assign a PSI > 40% in ~17% of the protein pairs.

Next, we have analyzed the relative improvement in the TM-score by Fr-TM-align. The TM-score difference (dTM) is defined as (TM-score (Fr-TM-align) – TM-score (TM-align)). The exact value of dTM which shows a significant change in the alignment quality is difficult to quantify. In fact, the numerical value of the TM-score has a contribution from the mean square distance between residues as well as from the number of aligned residues. The origin of a given dTM value depends on the TM-score as well. For example, an increase of 0.05 (dTM) in the TM-score from the initial value of 0.85 most likely arises because alignments with lower RMSD at identical coverage are generated. Whereas given an initial TM-score of 0.45, the increase to 0.5 most likely reflects both a decrease in RMSD and an increase in alignment coverage. Since most TM-scores of interest (as we are interested in detecting more distant similarities), are below 0.6, we have empirically considered a dTM ≥ 0.05 as a significant improvement and a dTM between 0 and 0.05 as not so significant only for the heuristic comparison of performance of TM-align with Fr-TM-align.

**Figure 4:**
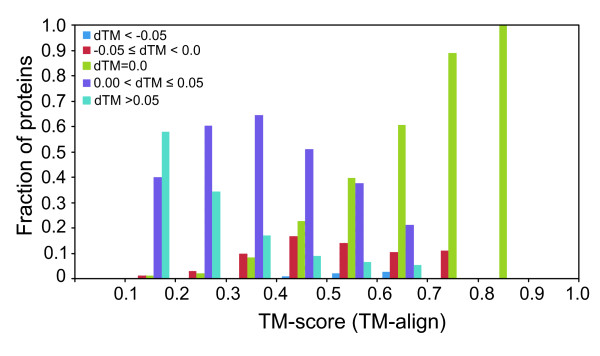
**Histogram showing the fraction of protein pairs with improved/decreased or unchanged TM-score by Fr-TM-align with respect to the TM-score reported by TM-align.** dTM is defined as (TM-score (Fr-TM-align) – TM-score (TM-align)).

**Figure 5:**
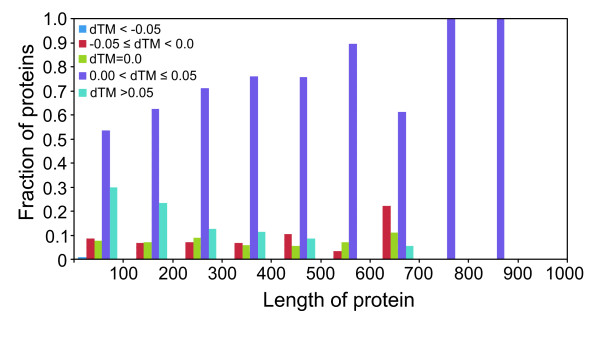
**Histogram showing the fraction of protein pairs with improved/decreased or without any change in TM-score by Fr-TM-align with respect to the length of the smaller protein of the two proteins being aligned.** dTM is defined as (TM-score (FrTMalign) – TM-score (TM-align)).

We present the results below only for dataset 2. Using this criterion (dTM ≥ 0.05), Fr-TM-align shows improvements in TM-score for ~28% of protein pairs being compared. Of the 19,900 pairs, ~56% of protein pairs have a dTM between 0.0 and 0.05, and for ~8%, the TM-score remains unchanged. We have also assessed the correlation between dTM and the TM-score from TM-align, to see if Fr-TM-align results in improved TM-scores for only a range of TM-score as reported from TM-align. dTM is divided into 5 bins (dTM < -0.05; -0.05 ≤ dTM < 0.00; dTM = 0.0; 0.00 < dTM ≤ 0.05 and dTM > 0.05). For a bin size of 0.1 of TM-score as reported from TM-align, we calculated the fraction of proteins in the above mentioned dTM bins. Figure 4 shows the fraction of proteins in each dTM bin for the range of TM-scores. It is evident that Fr-TM-align clearly improves the TM-score over all the ranges of TM-score as reported from TM-align. It is interesting to note that significant improvement (dTM > 0.05) is observed for more protein pairs in the lower ranges of TM-score. It was previously reported that using TM-align, the average TM-score for a randomly related protein structures is 0.30 [34]. In our study, of the 40% of protein pairs in the range of TM-score (0.2–0.3), ~35% of pairs of protein pairs show an improvement of more than 0.05 in TM-score by Fr-TM-align. This suggests that many pairs, which previously were defined as randomly related by TM-align, now have a significant TM-score. Next, we evaluated the dependence of dTM on the length of the proteins being aligned. Figure 5 shows the histogram of the fraction of proteins in various dTM bins versus the length of the smaller of the two proteins. It is evident from Figure 5 that improvement in TM-score by Fr-TM-align is not strongly dependent on the lengths of the structurally aligned proteins.

**Figure 6:**
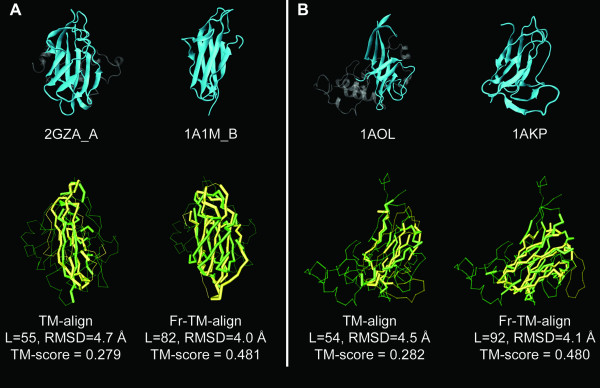
**Two examples showing the structural alignments from Fr-TM-align and TM-align.** A) The structural alignment between 2GZQ_A (186 residues) and 1A1M_B (99 residues). B) The structural alignment between 1AOL (228 residues) and 1AKP (114 residues). The first row shows the ribbon diagrams of the native structures. The beta-sheets in the native structures are colored in cyan to highlight the structurally similar region, while the remainder of the structure is transparent gray. The second row is the structural alignment given by TM-align and Fr-TM-align. L denotes the number of aligned residues. The longer of the two proteins is shown in light green color and the smaller protein is shown in light yellow color. The aligned (unaligned) regions are shown in the thick backbone (thin) backbone.

In Figures 6A and 6B, we show illustrative examples of the improvement in structure alignments when Fr-TM-align is used. Figure 6A shows the structural alignment between 1A1M_B and 2GZQ_A, which have a common β-sheet core. Fr-TM-align is able to align the common β-sheet; in addition, it is able to extend the alignment to include another two β-stands relative to the alignment obtained from TM-align. Figure 6B shows the alignment between 1AOL and 1AKP, which have β-strand regions in common. In comparison to the structural alignment from TM-align, Fr-TM-align extends the region of structural similarity in the common region of β-strands, which results in a better TM-score value.

On average, Fr-TM-align takes ~4.5 seconds of CPU time per structure pair on a single core of a 2 GHz AMD Opteron processor. This is ~12 times slower than TM-align. The speed of the program might limit its application for performing structural alignments on a very large scale, but with the increase in the number of cores/processor, this is not likely to be a practical impediment. To increase the speed of Fr-TM-align, we have implemented a slightly fast version of the algorithm, which essentially scans a smaller number of initial seed alignments with the heuristic iteration (as discussed in Methods). This is provided as an option before the execution of the program. On average, for all structure pairs compared, Fr-TM-align with the fast option takes ~2.8 seconds with a final average (standard deviation) in the TM-score of 0.277 (0.093). This is comparable to the average (standard deviation) TM-score, 0.279 (0.094), obtained using the slower and more sensitive version of Fr-TM-align. The faster version of the algorithm results in a TM-score improvement of ~8.6% in comparison to TM-align and is ~7 times slower. This could potentially be used for large scale scanning to detect remote structural similarities. The slower version provides slightly better structural alignments for some structure pairs. The source code and executables of Fr-TM-align are available at: .

## Conclusion

We have developed an improved structural alignment algorithm Fr-TM-align that uses a fragment alignment and assembly approach to generate initial seed alignments which when refined generates more significant structural alignments than those provided by the original TM-align program. We have compared Fr-TM-align with other competing structural alignment algorithms, TM-align and CE, using various alignment quality assessment scores such as PSI and the TM-score. The evaluation shows that Fr-TM-align performs better in comparison to both TM-align and CE. In comparison to TM-align, on an average, Fr-TM-align results in an improved TM-score (by ~9%) and increased coverage (by ~7%) in comparison to TM-align. A more detailed comparison between Fr-TM-align and TM-align shows that alignments from Fr-TM-align have an improved TM-score for ~86% of protein pairs. The improvement in TM-score by Fr-TM-align is observed for all lengths of protein pairs and over all TM-score (obtained from TM-align) ranges. However, Fr-TM-align achieves this higher accuracy and coverage at the expense of longer computation time. On an average, Fr-TM-align is ~12 times slower than TM-align. Nevertheless, the ability of Fr-TM-align to detect more subtle structural similarities is a more desirable attribute. For a more practical application of Fr-TM-align, we have modified the algorithm that has reduced the computation time by ~1.6 times with a decrease in final TM-score only by ~1% relative to the slower version of the algorithm.

## Authors' contributions

SBP participated in the design of the study and implemented the algorithm. JS conceived of the study, participated in its design and coordination and helped to draft the manuscript. All authors read and approved the final manuscript.
